# Hyperdynamic CSF motion profiles found in idiopathic normal pressure hydrocephalus and Alzheimer’s disease assessed by fluid mechanics derived from magnetic resonance images

**DOI:** 10.1186/s12987-017-0077-y

**Published:** 2017-10-18

**Authors:** Ken Takizawa, Mitsunori Matsumae, Naokazu Hayashi, Akihiro Hirayama, Satoshi Yatsushiro, Kagayaki Kuroda

**Affiliations:** 1 Department of Neurosurgery, Tokai University School of Medicine, 143 Shimokasuya, Isehara, Kanagawa 2591193 Japan; 2 Course of Science and Technology, Graduate School of Science and Technology, Tokai University, 4-1-1 Kitakaname, Hiratsuka, Kanagawa 2591292 Japan; 3 Department of Biological Engineering, Tokai University, School of Biological Engineering, 4-1-1 Kitakaname, Hiratsuka, Kanagawa 2591292 Japan

**Keywords:** Idiopathic normal pressure hydrocephalus, Alzheimer’s disease, Cerebrospinal fluid, Magnetic resonance imaging, Fluid dynamics

## Abstract

**Background:**

Magnetic resonance imaging (MRI) does not only ascertain morphological features, but also measures physiological properties such as fluid velocity or pressure gradient. The purpose of this study was to investigate cerebrospinal fluid (CSF) dynamics in patients with morphological abnormalities such as enlarged brain ventricles and subarachnoid spaces. We used a time-resolved three dimensional phase contrast (3D-PC) MRI technique to quantitatively evaluate CSF dynamics in the Sylvian aqueduct of healthy elderly individuals and patients with either idiopathic normal pressure hydrocephalus (iNPH) or Alzheimer’s disease (AD) presenting with ventricular enlargement.

**Methods:**

Nineteen healthy elderly individuals, ten iNPH patients, and seven AD patients (all subjects ≥ 60 years old) were retrospectively evaluated 3D-PC MRI. The CSF velocity, pressure gradient, and rotation in the Sylvian aqueduct were quantified and compared between the three groups using Kolmogorov–Smirnov and Mann–Whitney U tests.

**Results:**

There was no statistically significant difference in velocity among the three groups. The pressure gradient was not significantly different between the iNPH and AD groups, but was significantly different between the iNPH group and the healthy controls (p < 0.001), and similarly, between the AD group and the healthy controls (p < 0.001). Rotation was not significantly different between the iNPH and AD groups, but was significantly different between the iNPH group and healthy controls (p < 0.001), and similarly, between the AD group and the healthy controls (p < 0.001).

**Conclusions:**

Quantitative analysis of CSF dynamics with time resolved 3D-PC MRI revealed differences and similarities in the Sylvian aqueduct between healthy elderly individuals, iNPH patients, and AD patients. The results showed that CSF motion is in a hyperdynamic state in both iNPH and AD patient groups compared to healthy elderly individuals, and that iNPH patients and AD patients display similar CSF motion profiles.

## Background

The Cerebrospinal fluid (CSF) removes unnecessary substances and heat produced by metabolic activity from the brain parenchyma [1]. Therefore, understanding CSF dynamics is important for evaluating intracranial changes, pathological analysis, and treatment management of idiopathic normal pressure hydrocephalus (iNPH) and Alzheimer’s disease (AD) patients. Lately, noninvasive techniques using magnetic resonance imaging (MRI) have become prevalent in the analysis of CSF motion dynamics, and researchers commonly use phase contrast (PC) MR images to understand CSF physiology [2–6]. In the present study, we analyzed CSF motion using PC. Specifically, we used a time-resolved three-dimensional PC (3D-PC) MRI acquisition where the time dimension is added to the anterior–posterior, right–left, and head-foot dimensions in order to quantify and analyze CSF motion in elderly healthy individuals, idiopathic normal pressure hydrocephalus (iNPH) patients, and Alzheimer’s disease (AD) patients. In two-dimensional imaging, CSF velocity can only be calculated in-plane. Using 3D-PC, CSF motion can be calculated in three dimensions, and therefore the pressure gradient and rotation of the CSF motion can be measured in addition to velocity. Understanding fluid dynamics is important to determine the pressure gradient and rotation. CSF motion in elderly groups has only been investigated in a small number of studies and there is no consensus on the characteristics of CSF motion in AD patients [3, 7, 8]. In the iNPH study, CSF motion appears to be hyper dynamic [3, 9, 10]. The aim of this study was to compare the CSF dynamics of healthy elderly volunteers with those of AD and iNPH patients.

## Methods

This study was conducted with informed consent from volunteers and patients after explaining the purpose of the study in accordance with the ethics regulations of the authors’ affiliated institution.

### Patients

The patient’s characteristics are shown in Table 1. The subjects included seven AD patients (age range 66–89 years), ten iNPH patients (age range 67–87 years), and nineteen healthy elderly volunteers (age range 67–80 years). The iNPH group was selected in accordance with the guidelines outlined by the Japanese Society of Normal Pressure Hydrocephalus [11] and consisted of patients who displayed at least one symptom of the classical medical triad (gait disturbance, cognitive dysfunction, or urinary incontinence) and presented an enlargement of the anterior lateral ventricle horn with an Evans ratio ≥ 0.3, narrowing of high convexity subarachnoid space, and enlargement of the Sylvian fissure [12]. Eight out of ten iNPH patients received a shunting procedure: six received a ventriculo-peritoneal shunt and two patients a lumbo-peritoneal shunt. Gait disturbance improved in all patients, cognitive function improved in four patients, and urinary dysfunction improved in six patients after the shunting procedure. The AD group included individuals with probable AD according to the criteria delineated by the National Institute of Neurological and Communicative Diseases and Stroke/Alzheimer’s Disease and Related Disorders Association [13]. The healthy elderly control group was selected from volunteers recruited at our institution that did not have a history of central nervous system disorders, did not exhibit neurological abnormalities upon examination by neurologists or neurosurgeons, and did not present abnormalities on neurological and standard MRI.

**Table 1:** Characteristics of the group

	iNPH	AD	Control
Number of subjects	10	7	19
Mean age	73	77	74
Age range	67–87	66–89	67–80
Female	5	5	12

### MRI acquisition

A 1.5 Tesla MRI scanner with an eight-channel phased array head and neck surface coil (Gyroscan; Philips, Best, Netherlands) was used. A time resolved 3D-PC MRI sequence was used, providing a 4D velocity field. Flow-encoding directions were head–foot, right–left, and anterior–posterior. A dynamic cine image of one cardiac cycle was created by imaging 32 phases per heartbeat (without interpolation), synchronized with the heartbeat. The imaging parameters were: repetition time, 9.8–16.4 ms; echo time, 6.6–6.7 ms; flip angle, 20°; field of view, 22 × 22 for females and 32 × 32 cm^2^ for males; velocity encoding, 5 cm/s for volunteers, 30 cm/s for AD and iNPH patients; acquisition matrix, 1.96 × 1.96 × 1.96 mm^3^ (isotropic); sensitivity encoding factor of 2. Ten slices per volume were acquired, using 4–8 pixels to calculate the velocity and pressure gradient, and 12–20 pixels for the rotation. The acquisition time for this sequence was on average 32 min, depending on heart rate. The trigger for timing the 3D-PC was the peripheral pulse, measured from a finger.

### Image processing

Pressure gradients were calculated from the velocity data using Navier–Stokes equations. Color-coded CSF velocity vectors, pressure gradients, and rotations were overlaid on T_2_-weighted images. All processing was performed on a workstation (Power Mac Pro, Quad-Core Intel Xeon; Apple Inc., Cupertino, CA, USA) with our in-house software, written in Matlab (R2012b; Mathworks, Natick, MA, USA). Regions of interest (ROIs) at both the entry point and outlet point of the Sylvian aqueduct were drawn on sagittal images by the researchers, including a neurosurgery specialist, using an in-house mouse-operated graphical user interface. Partial volume effects caused by the relatively large voxel size (approximately 2 mm) used can lead to segmentation errors on T_2_-weighted images. Therefore, a “spatial-based fuzzy clustering” segmentation method [14] was used to reduce partial volume effects.

The principle of this study was based on obtaining velocity and rotation in three-dimensional space (anterior–posterior, right–left, and head–foot directions) using the PC technique. The pressure gradient was then calculated from these velocity data using Navier–Stokes equations. Further details of this method can be found elsewhere [4].

### Statistical analysis

CSF velocity and pressure gradient data calculated from the ROIs were represented as box plots. CSF velocities and pressure gradients were compared between groups as nonparametric data using Kolmogorov–Smirnov and Mann–Whitney U tests. All statistical analyses were conducted using SPSS software version 13 (SPSS Japan Inc., Tokyo).

## Results

### Comparison of velocity between AD patients, iNPH patients, and healthy elderly volunteers

**Fig. 1:**
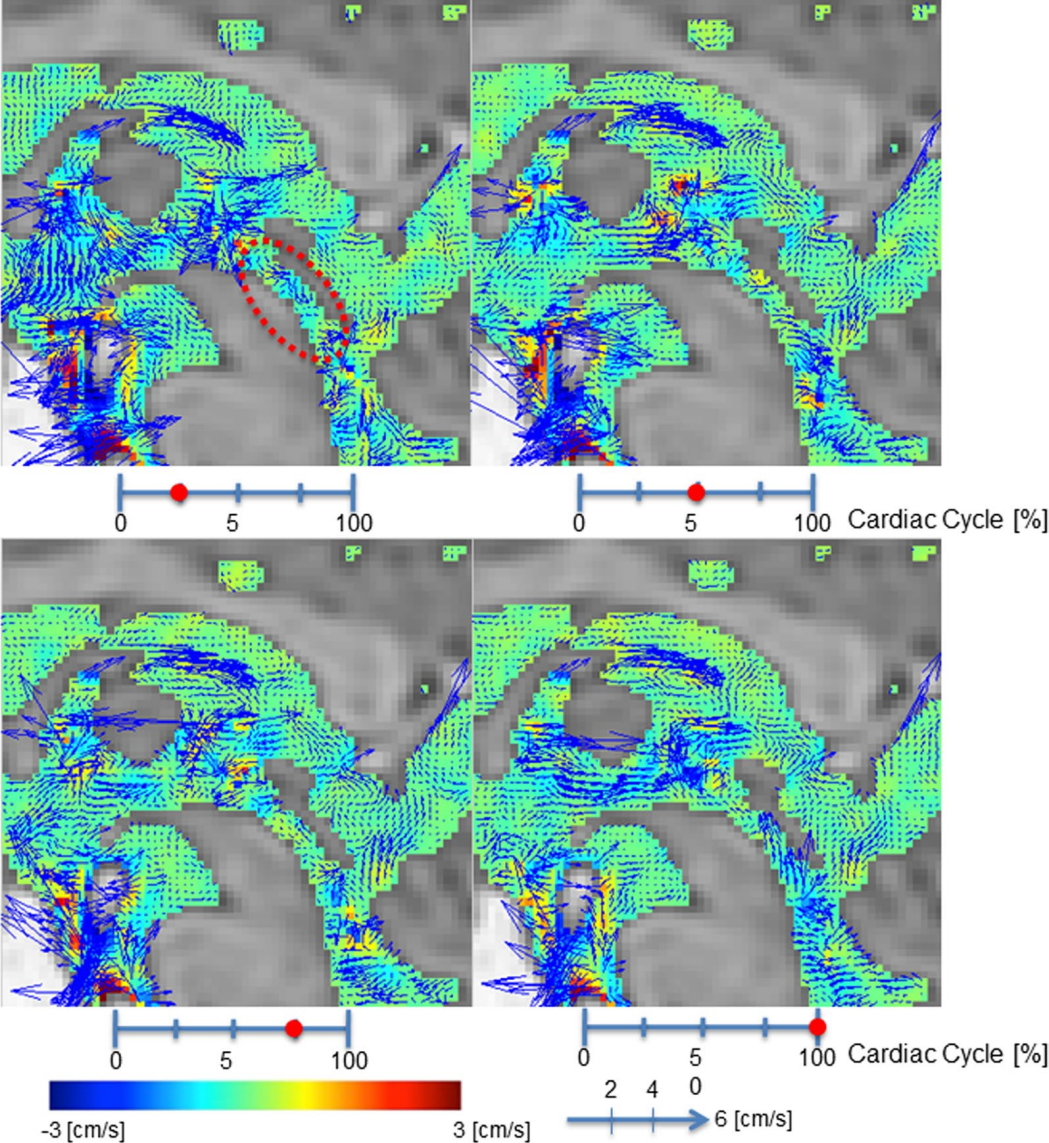
Velocity mapping of the volunteer by 3D-PC at 4 different stages of the cardiac cycle. In-plane velocities were visualized using vectors and through-plane velocities were visualized using colors. The color-coded CSF velocity field vector was then superimposed onto T_2_-weighted images of the stationary tissues. Red circle indicates Sylvian aqueduct

**Fig. 2:**
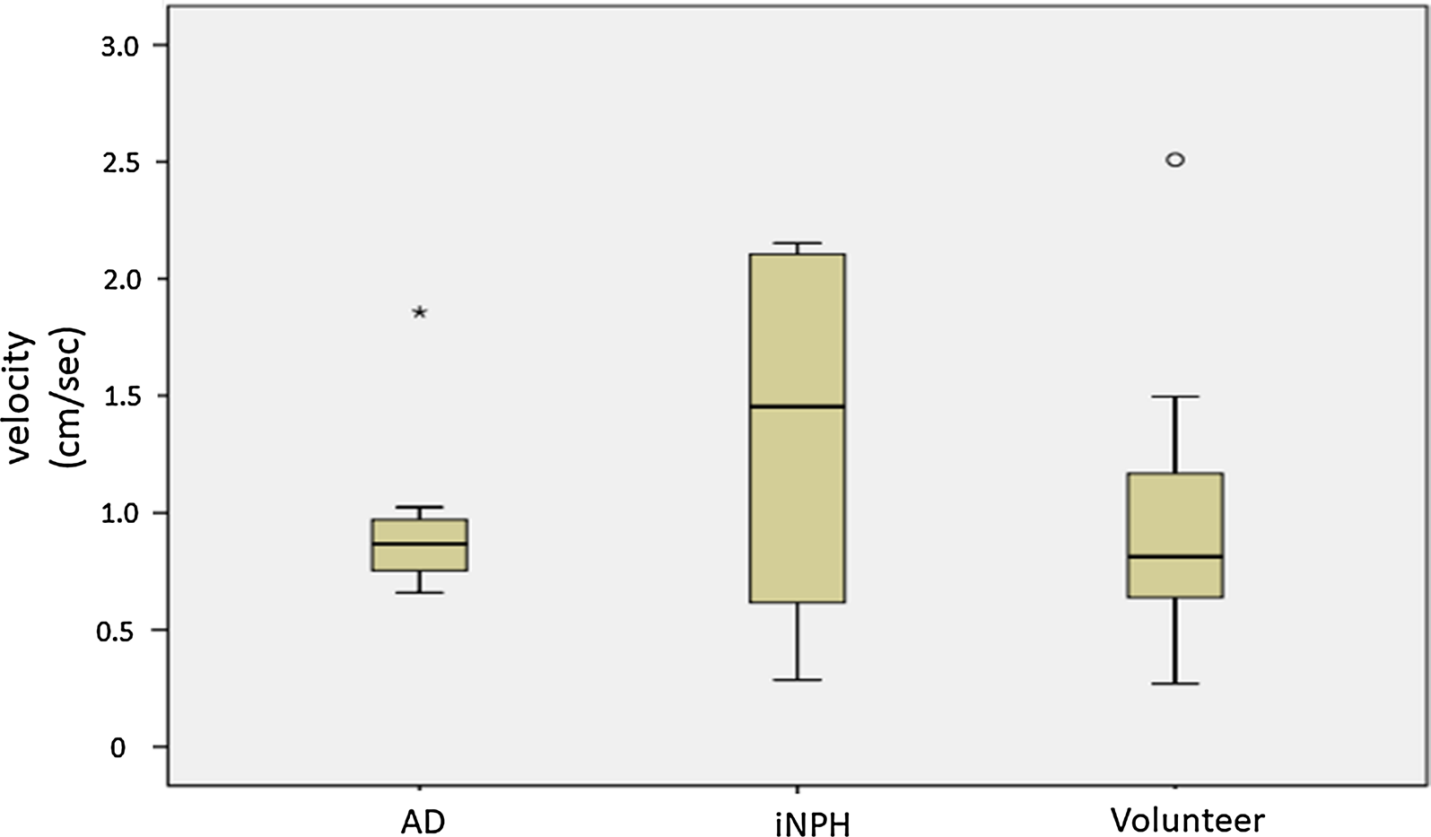
Box plots of velocity in the Sylvian aqueduct for AD patients, iNPH patients, and healthy elderly volunteers. There were no statistically significant differences between the patient groups and volunteer group. AD Alzheimer’s disease, iNPH idiopathic normal pressure hydrocephalus outside values are indicated by a small “o” and far-out values are indicated by an asterisk

Figure 1 shows velocity images for a 67 year old male volunteer. Figure 2 shows the calculated velocity in the Sylvian aqueduct of AD patients, iNPH patients, and healthy elderly volunteers. There was no statistically significant difference (p = 0.380) between the AD (median = 0.868 cm/s) and iNPH groups (median = 1.452 cm/s). Similarly, there were no significant differences (p = 0.912) between the AD and healthy controls (median = 0.801 cm/s) or between the iNPH and healthy controls (p = 0.271).

### Comparison of pressure gradient between AD patients, iNPH patients, and healthy elderly volunteers

**Fig. 3:**
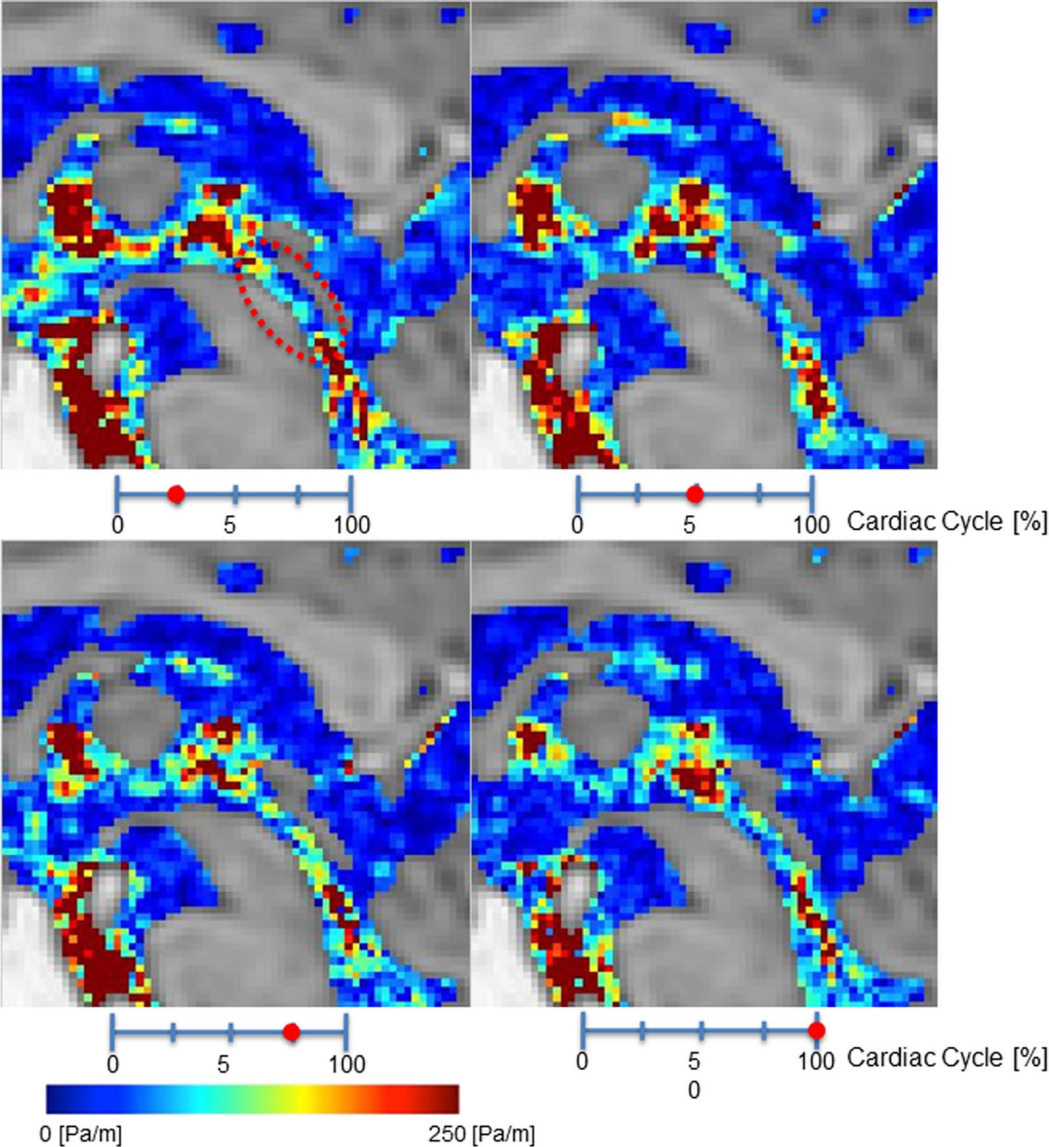
Pressure gradient color mapping of the volunteer by 3D-PC at 4 stages of the cardiac cycle. The intensity of the pressure gradient was indicated using a color scale. The color-coded CSF pressure gradient were then superimposed onto T_2_-weighted images of the stationary tissues. Red circle indicates Sylvian aqueduct

**Fig. 4:**
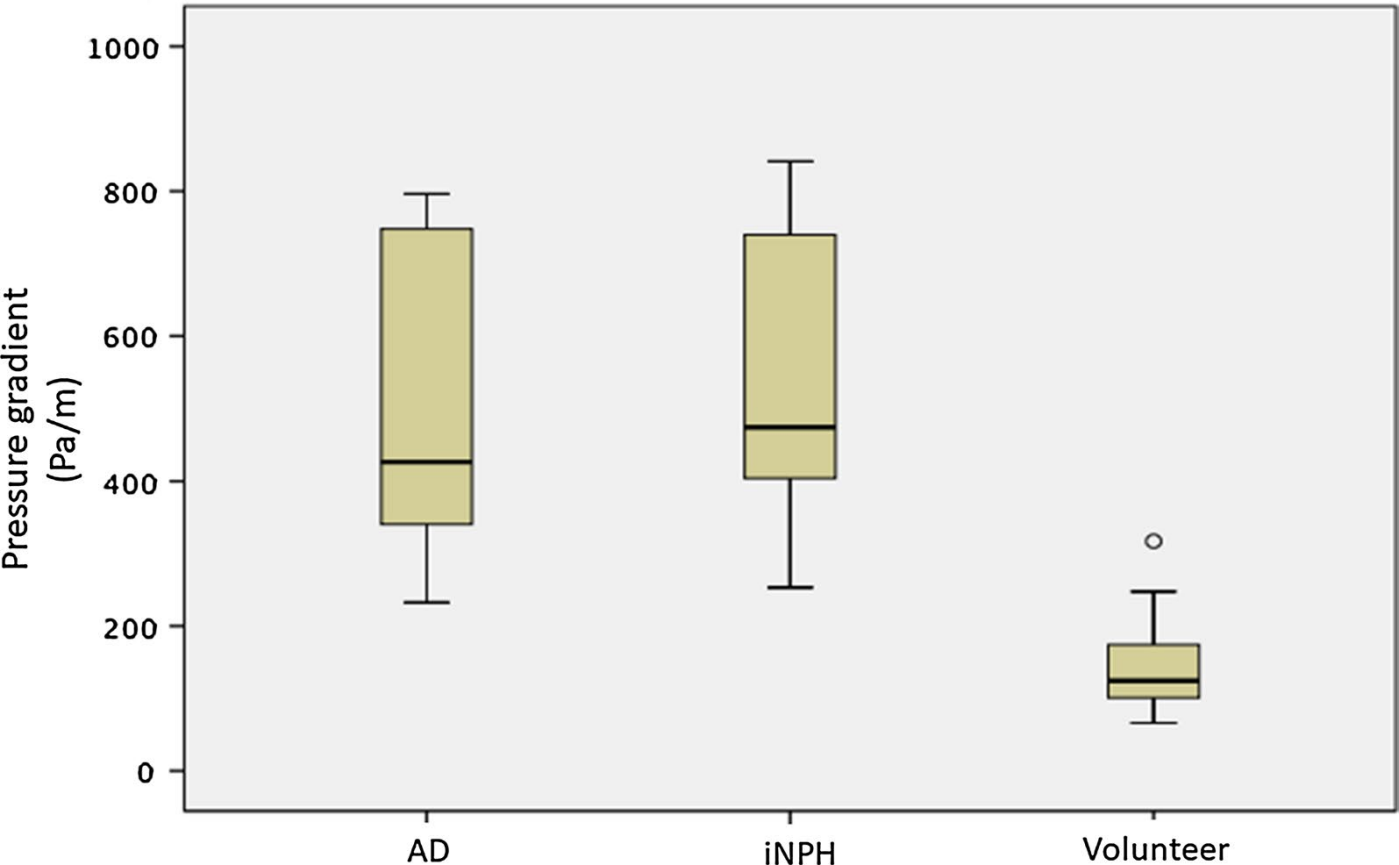
Box plots of pressure gradients in the Sylvian aqueduct between AD patients, iNPH patients, and healthy elderly volunteers. Both the AD and iNPH groups had significantly greater pressure gradients compared to the volunteer group. AD Alzheimer’s disease, iNPH idiopathic normal pressure hydrocephalus outside values are indicated by a small “o”

Figure 3 shows pressure gradient images for a 67-year-old male volunteer. Figure 4 shows the pressure gradient in the Sylvian aqueduct of AD patients, iNPH patients, and healthy elderly volunteers. The median pressure gradients for each group were: 426.6 Pa/m for the AD group, 473.8 Pa/m for iNPH group, and 117.8 Pa/m for the healthy elderly group. Both the AD and iNPH groups had significantly greater pressure gradients in the Sylvian aqueduct compared to the healthy controls (p < 0.001 for both comparisons). There was no significant difference between the AD and iNPH groups (p = 0.696).

### Comparison of rotation between AD patients, iNPH patients, and healthy elderly volunteers

**Fig. 5:**
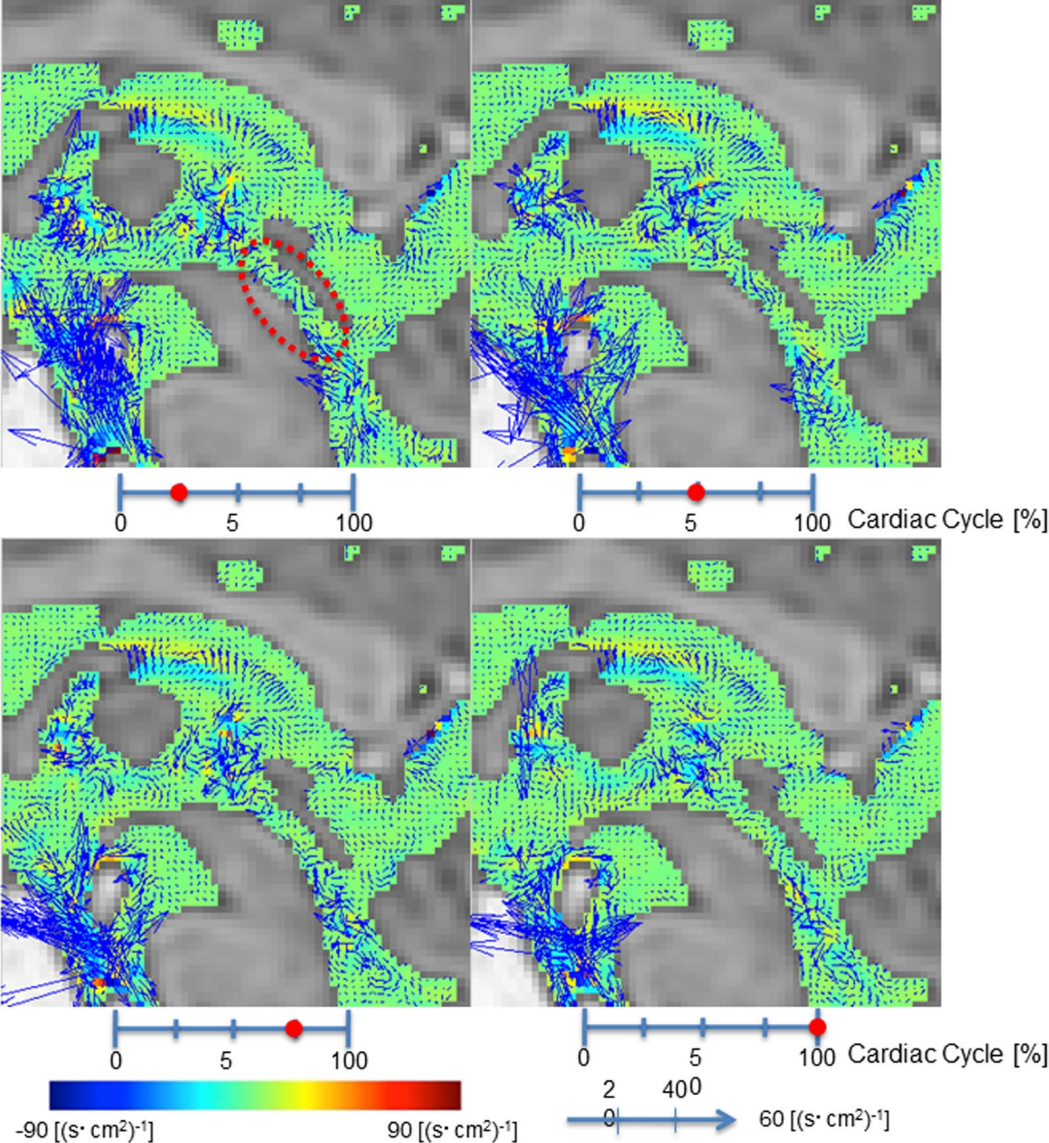
Rotation mapping of the volunteer by 3D-PC at 4 stages of the cardiac cycle. In-plane rotations were visualized using vectors and through-plane rotations were visualized using colors. The color-coded CSF rotation field vector was then superimposed onto T_2_-weighted images of the stationary tissues. Red circle indicates Sylvian aqueduct

**Fig. 6:**
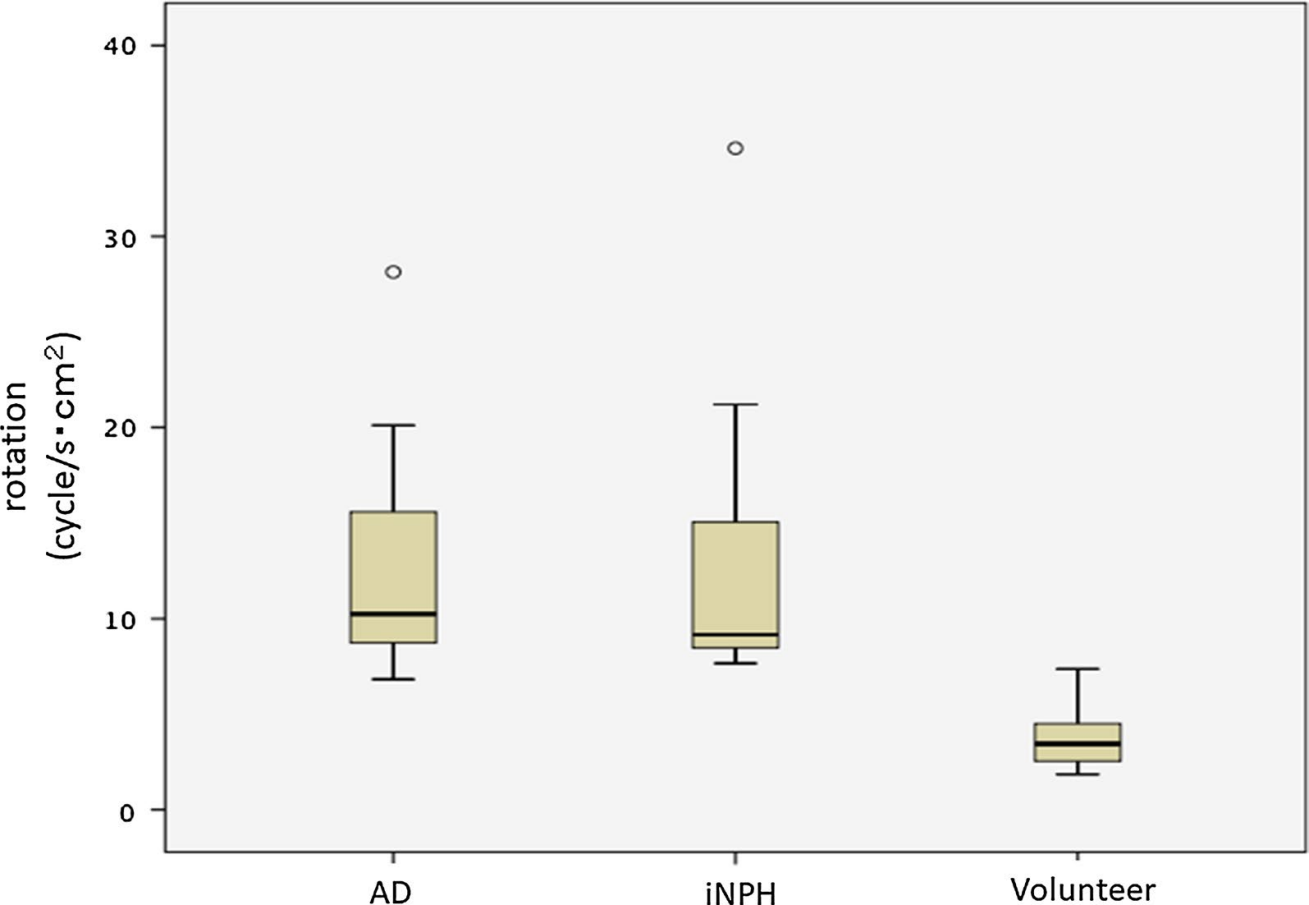
Box plots of rotation in the Sylvian aqueduct between AD patients, iNPH patients, and healthy elderly volunteers. Both the AD and iNPH groups had greater rotation compared to the volunteer group. AD: Alzheimer’s disease, iNPH idiopathic normal pressure hydrocephalus outside values are indicated by a small “o”

Figure 5 shows rotation images for a 67-year-old male volunteer. Figure 6 shows the rotation in the Sylvian aqueduct of AD patients, iNPH patients, and healthy elderly volunteers. There was no significant difference (p = 0.845) between the AD (median = 10.243 cycle/s cm^2^) and iNPH groups (median = 9.159 cycle/s cm^2^). Both the AD and iNPH groups had greater rotation compared to the healthy elderly group (median = 3.447 cycle/s cm^2^, p < 0.001 for both comparisons).

## Discussion

In the present study, we assessed three elements that describe CSF motion (CSF velocity, pressure gradient, and rotation) in the Sylvian aqueduct, using a time resolved 3D-PC MRI sequence and compared these between AD patients, iNPH patients, and healthy elderly volunteers. Although previous studies have investigated the physiology and pathology of AD and iNPH patients, few reports have analyzed CSF dynamics using MR, particularly in AD patients. Moreover, apart from one preliminary study [3], no other studies have shown that the pressure gradient is elevated in iNPH patients compared to healthy elderly controls, and our present report is the first to investigate the pressure gradient in AD patients. Therefore, the present study analyzed the components of fluid dynamics such as velocity, pressure gradient, and rotation, in order to better understand the pathology of a disease that also presents as a similar enlargement of CSF spaces.

The results showed that CSF velocity was not statistically significantly different between the AD and iNPH patient groups and healthy elderly volunteers. However, pressure gradient and rotation were significantly greater in AD and iNPH patient groups compared to the healthy elderly group. Pressure gradient was determined using the Navier–Stokes equations, consisting of acceleration, convection, and viscosity terms. We hypothesized that CSF viscosity and convection, as well as the anatomical morphology of the Sylvian aqueduct are not significantly different between the AD and iNPH patient groups and the healthy elderly group. This would mean that only acceleration has a significant effect on the pressure gradients of CSF, with acceleration increases resulting in pressure gradient increases. In other words, the AD and iNPH patient groups had greater CSF flow acceleration compared to the healthy controls, and this consequently influenced the pressure gradient. Furthermore, large velocity changes may lead to a disturbance in CSF motion, inducing turbulence in the Sylvian aqueduct, and this may have also been reflected in the large differences in rotation. Thus, it appears that CSF dynamics are in a hyper-dynamic state in both the AD and iNPH patient groups. The details of the relationship between pressure gradient and or velocity compared with rotation is described elsewhere [15]. To the best of our knowledge, this is the first study to report CSF rotation in AD and iNPH patients. Moreover, apart from one preliminary study [3], no other studies have shown that the pressure gradient is elevated in iNPH patients compared to healthy elderly controls, and our present report is the first to investigate the pressure gradient in AD patients. On the other hand, there are various PC MRI studies that have shown that the CSF flow in iNPH patients is in a hyper-dynamic state, which is in accordance with our results. Specifically, it has been shown that mean CSF flow is greater in iNPH but not AD patients compared to healthy controls [7, 10], CSF motion increases in iNPH patients [16], and stroke volume increases in iNPH patients [9, 17, 18]. However, it is not currently possible to use markers such as stroke volume [8] to distinguish between iNPH and AD. Also, we have compared the result of preoperative CSF dynamics and postoperative surgical results in iNPH group. Due to the limited number of patients, we currently could not differentiate between shunt responders and shunt non-responders.

These results pose the question as to why CSF is in a hyper-dynamic state in our patients compared to the healthy elderly group. A decrease in brain volume due to aging or AD leads to a relative increase in ventricular volume and subarachnoid space leading to a general increase in CSF volume, and this consequently increases the space available for free CSF motion. It is unlikely that this increased freedom leads to pressure gradient elevation, rotation increase, or turbulence. On the contrary, it would most likely decrease pressure gradient and rotation. If so, the most probable factor inducing elevated CSF pressure gradients and increased rotation would be the restriction in CSF motion due to decreased compliance in the cerebrospinal cavity that surrounds the subarachnoid space and ventricles. Mase and colleagues reported that iNPH patients had lower compliance compared to patients with ventricular dilatation or asymptomatic ventricular dilatation and healthy volunteers [19–21]. On the other hand, Edwards et al. described that the compliance of spinal subarachnoid space (especially dural sac) has a large effect on the compliance of overall CSF, indicating that degenerative diseases of the spinal cord induce decreased compliance [22]. Studies using mathematical models showed that decreased compliance is a significant element in ventricle enlargement [23, 24], and simulations using these models are extremely easy to understand. These results all indicated that decreased compliance in the CSF environment is important in diseases such as iNPH that are associated with enlarged ventricles. Furthermore, Bateman et al. investigated compliance in iNPH and AD groups and found that iNPH patients had significantly lower compliance compared to healthy volunteers and that the AD group had a compliance that was midway between the healthy volunteers and the iNPH group [25], corroborating the presence of decreased compliance in iNPH patients and suggesting that further investigations are required for AD patients. Based on these reports, it seems likely that the increased CSF pressure gradient and rotation in the Sylvian aqueduct of iNPH patients are due to decreased compliance in the cerebrospinal cavity. This is presumably the case in AD patients who also exhibit increased CSF pressure gradients and rotation in the Sylvian aqueduct.

In the above section, we discussed the common pathological state between iNPH and AD based on the fluid dynamics analysis of CSF. Regarding the discrimination of iNPH and AD, there are some contradictory findings in brain tissue biopsy [26, 27] and examinations using CSF biomarkers may not be able to differentiate between these groups [28]. This indicated that there are overlaps between iNPH and AD in addition to the pathological condition assessed based on the fluid dynamics analysis of CSF we reported in the present study. Therefore, it can be concluded that there is not currently a suitable method to distinctly classify iNPH and AD.

A limitation of the present technique is that this quantitative measurements based on the PC technique can only measure fluid velocity with 10% accuracy [29], especially for small voxel size.

## Conclusions

In the present study, we quantitatively analyzed time-resolved 3D-PC MRI data of the Sylvian aqueduct in iNPH and AD patients, and healthy elderly volunteers. The 3D-PC method is useful to understand CSF dynamics in AD, iNPH, and healthy elderly volunteers. As iNPH and AD patients show a different CSF motion profile from that of healthy elderly individuals, it suggests that there is a difference in compliance between the patient groups and healthy controls.

## Abbreviations

CSF: cerebrospinal fluid
MRI: magnetic resonance imaging
PC: phase contrast
3D-PC: three-dimensional phase contrast
iNPH: idiopathic normal pressure hydrocephalus
AD: Alzheimer’s disease
